# Premature Burial

**Published:** 1905-03-25

**Authors:** 


### Premature Burial.

The fear of death is one of the most deeply-planted instincts in the human race, but the fear of premature burial has at all times run it close. Both, like all instincts, are unreasoning, and often appear in the most unlikely minds, for they are met with in the highly cultured as well as in the densely ignorant. The fear of premature burial is a mere vestigium or remnant of an earlier stage of existence than any known to the Northern races of Europe, yet the fear is firmly implanted in the minds of its peoples. It is conceivable that such an accident might happen in the South of Europe and amongst the inhabitants of a climate which might make speedy burial a necessity. It has actually taken place on the battle-field from mere callousness, and during vast epidemics, which have disorganised the usual routine of life leading to burial in pits rather than in graves. It could not happen in modern civilised life where a body is kept for several days before it is committed to the earth or is burnt. In the rare cases where a person has recovered consciousness whilst preparations were being made for interment it has always been before the closure of the coffin and whilst there was free access of air. The closure of the coffin with the consequent deprivation of air would infallibly quench the dormant spark of life within a very short time and if the patient were unconscious at the time of closure it is very certain that he would not afterwards recover consciousness, because the emanations from his own lungs and body in so confined a space would surely asphyxiate him and insensibility is amongst the earliest phenomena of suffocation. The stories told by irresponsible people without any possibility of verifying dates and places may be safely treated as untrue when they assert that persons have come to life again after the ordinary burial adopted here, in France, or in Germany. They may be looked upon as apocryphal and in the nature of jugglery when they narrate, as in the case of the Indian fakir, that he was hypnotised, buried for three weeks with an English sentry over him, and then exhumed, thinner than before, but otherwise fresh and hearty.

The earliest record of premature burial is said to date from the 84th Olympiad, or B.C. 444 in the time of Herodotus and during the golden age of Pericles when Empedocles restored to life a woman who was on the point of being buried. The affair was so noised abroad that a law was enacted amongst the Greeks to prevent the interment of anyone until the sixth or seventh day after death. Such a matter would naturally appeal to the æsthetic and neurotic Athenians. But the Romans were made of sterner stuff, and it was not until the reign time of Pompey about B.C. 49, when Greek culture had made great progress in Italy that a similar event led to a like result. It is told that a physician of that day watched a crowd of mourners preparing to set alight a funeral pyre. Looking at the body he thought he perceived some signs of life, he therefore ordered the pyre to be destroyed, and by diligent application resuscitated the body. Other cases of a similar nature occurring about the same time the matter of premature burial attracted public notice, and a law was passed directing that the last sad rites should not be performed until the eighth day after death. The Turks in later years had a similar horror of premature burial, and they are reported to have made use of the contractility of the sphincters as one of their most certain signs of death. In England there exists an association for the Prevention of Premature Burial, which seeks to obtain some amendments in the present law regulating burial. The object of the proposed Bill is to provide security against the burial of persons who are only apparently dead and who might recover if time were allowed. Provision is to be made for two independent medical opinions, for the establishment and remuneration of certain medical inspectors or "death verifiers," the provision of mortuaries where bodies may be kept until the fact of death is conclusively ascertained and the granting power to a justice of the peace to issue exhumation orders in place of the Home Secretary. It is also sought to modify the present form of certificate of death in such a manner as to render a medical examination imperative before it can be legally signed. Some alteration of the present system of death certification would probably meet the approval of every medical practitioner and increased mortuary accommodation is undoubtedly necessary in many parts of the country, but it is very doubtful whether any general establishment of "waiting mortuaries" would be sanctioned by the local governing bodies, and it is highly undesirable to delegate the power of exhumation to justices of the peace.

